# Different correlation patterns of cholinergic and GABAergic interneurons with striatal projection neurons

**DOI:** 10.3389/fnsys.2013.00047

**Published:** 2013-09-03

**Authors:** Avital Adler, Shiran Katabi, Inna Finkes, Yifat Prut, Hagai Bergman

**Affiliations:** ^1^Department of Medical Neurobiology, Institute of Medical Research Israel-Canada, The Hebrew University-Hadassah Medical SchoolJerusalem, Israel; ^2^The Interdisciplinary Center for Neural Computation, The Hebrew UniversityJerusalem, Israel; ^3^Edmond and Lily Safra Center for Brain Sciences, The Hebrew UniversityJerusalem, Israel

**Keywords:** striatum, interneurons, crosscorrealtion, physiology, spikes

## Abstract

The striatum is populated by a single projection neuron group, the medium spiny neurons (MSNs), and several groups of interneurons. Two of the electrophysiologically well-characterized striatal interneuron groups are the tonically active neurons (TANs), which are presumably cholinergic interneurons, and the fast spiking interneurons (FSIs), presumably parvalbumin (PV) expressing GABAergic interneurons. To better understand striatal processing it is thus crucial to define the functional relationship between MSNs and these interneurons in the awake and behaving animal. We used multiple electrodes and standard physiological methods to simultaneously record MSN spiking activity and the activity of TANs or FSIs from monkeys engaged in a classical conditioning paradigm. All three cell populations were highly responsive to the behavioral task. However, they displayed different average response profiles and a different degree of response synchronization (signal correlation). TANs displayed the most transient and synchronized response, MSNs the most diverse and sustained response and FSIs were in between on both parameters. We did not find evidence for direct monosynaptic connectivity between the MSNs and either the TANs or the FSIs. However, while the cross correlation histograms of TAN to MSN pairs were flat, those of FSI to MSN displayed positive asymmetrical broad peaks. The FSI-MSN correlogram profile implies that the spikes of MSNs follow those of FSIs and both are driven by a common, most likely cortical, input. Thus, the two populations of striatal interneurons are probably driven by different afferents and play complementary functional roles in the physiology of the striatal microcircuit.

## Introduction

The striatum is the primary input stage of the basal ganglia network. Its medium spiny projection neurons (MSNs) constitute the vast majority of striatal cells (Tepper et al., 2008). However, their activity and hence striatal output is thought to be highly affected by a proportionally small population of a-spiny interneurons (Kawaguchi et al., 1995; Kreitzer, 2009; Tepper et al., 2010; Gittis and Kreitzer, 2012). Two major groups of striatal interneurons have been extensively studied by *in vitro* and *in vivo* physiological methods: the fast spiking parvalbumin (PV) expressing GABAergic interneurons (FSIs; e.g., Berke, 2008; Tepper et al., 2010) and the tonically active cholinergic interneurons (TANs; e.g., Kimura et al., 1984; Aosaki et al., 1994; Graybiel et al., 1994; Morris et al., 2004).

GABAergic FSIs form powerful perisomatic synapses onto MSNs (Tepper et al., 2008). *In vitro* studies suggest that the GABAergic FSIs provide strong feed-forward inhibition that shapes the firing patterns of MSNs (Tepper et al., 2008; Gittis et al., 2010; Planert et al., 2010). On the other hand, there are no reports of studies of mono-synaptic interactions between TANs and MSNs (but see English et al., 2012, for recent evidence for di-synaptic interactions between TANs and MSNs). TANs probably cannot be simply characterized as having an excitatory or inhibitory effect on MSN activity; rather they are assumed to have a global modulatory effect (Oldenburg and Ding, 2011).

FSIs have been found to display high sensitivity to cortical input (Mallet et al., 2005) and may integrate information from diverse cortical areas (Parthasarathy and Graybiel, 1997). Their *in vivo* extracellular activity has been described mainly in rodents, and exhibits robust task-related responses in operant conditioning paradigms (Berke, 2011). However, although FSIs are coupled by gap junctions (Kita et al., 1990; Koos and Tepper, 1999), their *in vivo* activity was shown to be highly individualized (Berke, 2008; Schmitzer-Torbert and Redish, 2008). Similarly, correlation studies of the spiking activity of simultaneously recorded FSI-MSN pairs failed to find strong evidence for mono synaptic inhibition of MSN activity by the FSI (Sharott et al., 2009; Gage et al., 2010; Lansink et al., 2010). The *in vivo* activity of TANs has been amply investigated in behaving primates. In associative learning paradigms these cells pause their tonic firing for a 200–300 milliseconds in response to external stimuli that become associated with rewarding (and aversive) outcomes (Kimura et al., 1984; Apicella, 2002; Joshua et al., 2008). The TANs receive excitatory inputs from cortex and thalamus, and have been shown to increase their discharge in response to direct cortical stimulation (Sharott et al., 2012). Although TANs receive both cortical and thalamic innervations, the TAN characteristic pause response is probably driven by thalamic input (Matsumoto et al., 2001; Nanda et al., 2009; Ding et al., 2010; Schulz et al., 2011).

Both striatal interneuron cell types have been shown to be imperative to normal striatal functioning (Pisani et al., 2007; Gittis et al., 2011). This makes it crucial to define the functional (*in vivo*) relationship between their activity and the MSNs that mediate striatal output. We thus recorded and analyzed the simultaneous spiking activity of MSN–TAN or MSN–FSI pairs and used cross-correlation methods to identify the direct synaptic interactions and/or common input drives of these striatal projection—interneurons pairs.

## Methods

Two monkeys (*Macaque fascicularis*, G male, 4.5 kg; L female, 3 kg) were used in this study. Experimental protocols were conducted in accordance with the National Research Council's *Guide for the Care and Use of Laboratory Animals* and the Hebrew University guidelines for the use and care of laboratory animals in research. The experimental protocols were approved and supervised by the Institutional Animal Care and Use Committee (IACUC) of the Hebrew University and Hadassah Medical Center. The Hebrew University is an Association for Assessment and Accreditation of Laboratory Animal Care (AAALAC) internationally accredited institute. The behavioral paradigm, surgery procedures, data-recording, and analysis methods were described in previous manuscripts (Adler et al., 2012, 2013). Here we only describe in detail the methods not previously used.

### Behavioral task

**Figure 1:**
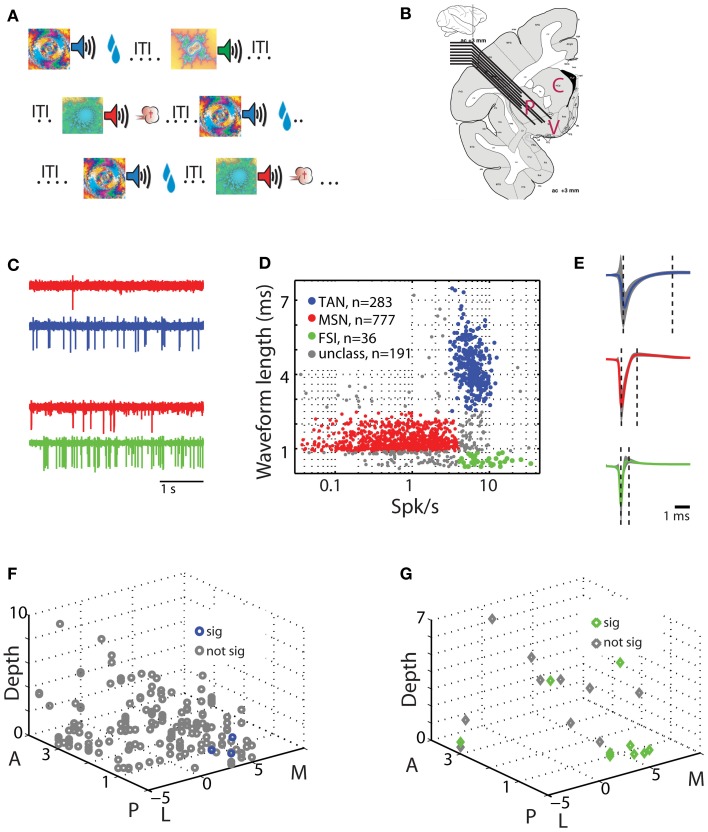
**Behavioral task and physiological recording methods. (A)** Classical conditioning paradigm. Visual cues were presented for 2 s and predicted the delivery of food (reward trials, upper row), air puff (aversive trials, third row), or only sound (neutral trials, second row). The trial outcome epoch was followed by a variable inter trial interval (ITI) of 5–6 s. **(B)** Recording sites: a representative coronal section +3 mm from the anterior commissure [adapted from Martin and Bowden, 2000]. Two to eight electrodes were advanced separately into one or two of the three sub regions of the striatum. P for putamen, C for caudate, and V for ventral striatum. **(C)** An example of simultaneously recorded pairs of units from the putamen. Each row is a 4 s analog trace of extracellular recording from a single electrode filtered between 300 and 6000 Hz. First two rows are MSN (red) and TAN (blue) recorded simultaneously, second two rows are MSN (red) and FSI (green) recorded simultaneously. **(D)** Classification of striatal neuron subtypes. Each dot represents a single neuron colored according to its subtype. Red, MSN; blue, TAN; green, FSI; gray, cells not categorized in either group. Abscissa: firing rate in Hz (logarithmic scale). Ordinate: spike waveform duration (ms). **(E)** Spike waveform averaged over all cells (average ± STD, line and shaded envelope, respectively) in each of the clusters. Waveform length was measured as the distance between the first negative peak and the next positive peak (left and right dashed lines, respectively). Upper row; TAN. Middle row: MSN. Third row: FSI. Same color coding as in **(D)**. **(F)** Spatial layout of TAN-MSN pairs. Each point represents a single pair. Abscissa: coordinates in the horizontal plane (in mm); M, medial; L, lateral; zero in the center of the putamen in our recordings. Ordinate: coordinates in the peri-sagittal plane (in mm); A, anterior; P, posterior; zero is coronal section AC0 (AC, anterior commissure). Z-axis: depth from entry to the striatum (in mm) of the TAN in the TAN-MSN pair. Blue and gray, location of pairs with significant and not significant correlations, respectively. **(G)** Spatial layout of FSI-MSN pairs. Same conventions as in **(F)**.

During recordings the monkeys were engaged in a well-practiced classical conditioning task involving rewarding, aversive, and neutral outcomes (Figure 1A). Details of the behavioral paradigm and monkey behaviors are provided in our previous reports (Adler et al., 2012, 2013). Briefly, each trial began with the presentation of a visual cue (full-screen fractal images) for a period of 2 s. The cues were immediately followed by an outcome which could be one of three categories: liquid food in the reward trials, air puff (directed at both eyes) in the aversive trials, or neither in the neutral trials. The beginning of the outcome epoch was signaled by one of three sounds (duration, 80 ms) that discriminated the three outcome categories. Trials were followed by a variable inter-trial interval (ITI) of 5–6 s. In each category there were three/two (monkey G and L, respectively) different visual cues. In the rewarding and aversive trials the cues were differentiated by the magnitude or intensity of the liquid food or air puff, respectively. In the neutral trials the cues were differentiated by a change in the duration of the ITI (−2/0/+2 s to ITI duration). In total there were nine/six (monkey G and L, respectively) different visual cues; three/two (monkey G and L, respectively) for each outcome category. In this study, we combined the trials within each outcome category and present the results for the rewarding trials (which include all amounts of liquid food), the aversive trials (which include all air puff intensities), and the neutral trials. Visual fractal cues and auditory sounds were randomized between monkeys.

Licking and blinking behavior was recorded by an infrared reflection detector (Dr. Bouis, Freiburg, Germany) and video computerized analysis (Mitelman et al., 2009). We have previously demonstrated (Adler et al., 2012, 2013) that during recordings the monkeys were familiar with the visual cues and displayed the appropriate anticipatory licking and blinking behavior. Specifically, they licked to the presentation of the rewarding (and not aversive or neutral) cues and they blinked to the presentation of the aversive (and not rewarding or neutral) cues.

### Recording and classification of extracellularly recorded striatal neurons

Striatal neuronal activity was recorded by two to eight glass coated tungsten microelectrodes (impedance at 1 KHz 0.3–0.8 Mohm and horizontal distance of 0.5 mm) that were advanced separately (EPS; Alpha-Omega Engineering) into the different domains of the anterior striatum (Figure 1B). The electrodes were slowly advanced in each recording session to enable optimal detection and sorting of the spontaneous spiking activity. We used two criteria to distinguish between striatal cell types: the cells' average firing rate, and extracellular spike waveform duration from the first negative peak to the following positive peak (Figures 1C–E). Cells with waveform durations of 0.9–2.5 ms and average firing rates <4 Hz were classified as presumed MSNs. Cells with waveform durations >2.5 ms and average firing rates of 3–15 Hz were classified as TANs. Finally, cells with waveform durations <0.9 ms and average firing rates >4 Hz were classified as FSIs. Remaining cells that did not strictly belong to the above groups were discarded (Figure 1D, unclassified) and are not reported here. Additional classifications using valley width at half maximum of the spike waveform and discharge pattern [coefficient of variation (CV) of the inter spike interval (ISI); see below] received similar identification (data not shown).

### Data analysis of the discharge pattern of striatal neurons

**Figure 2:**
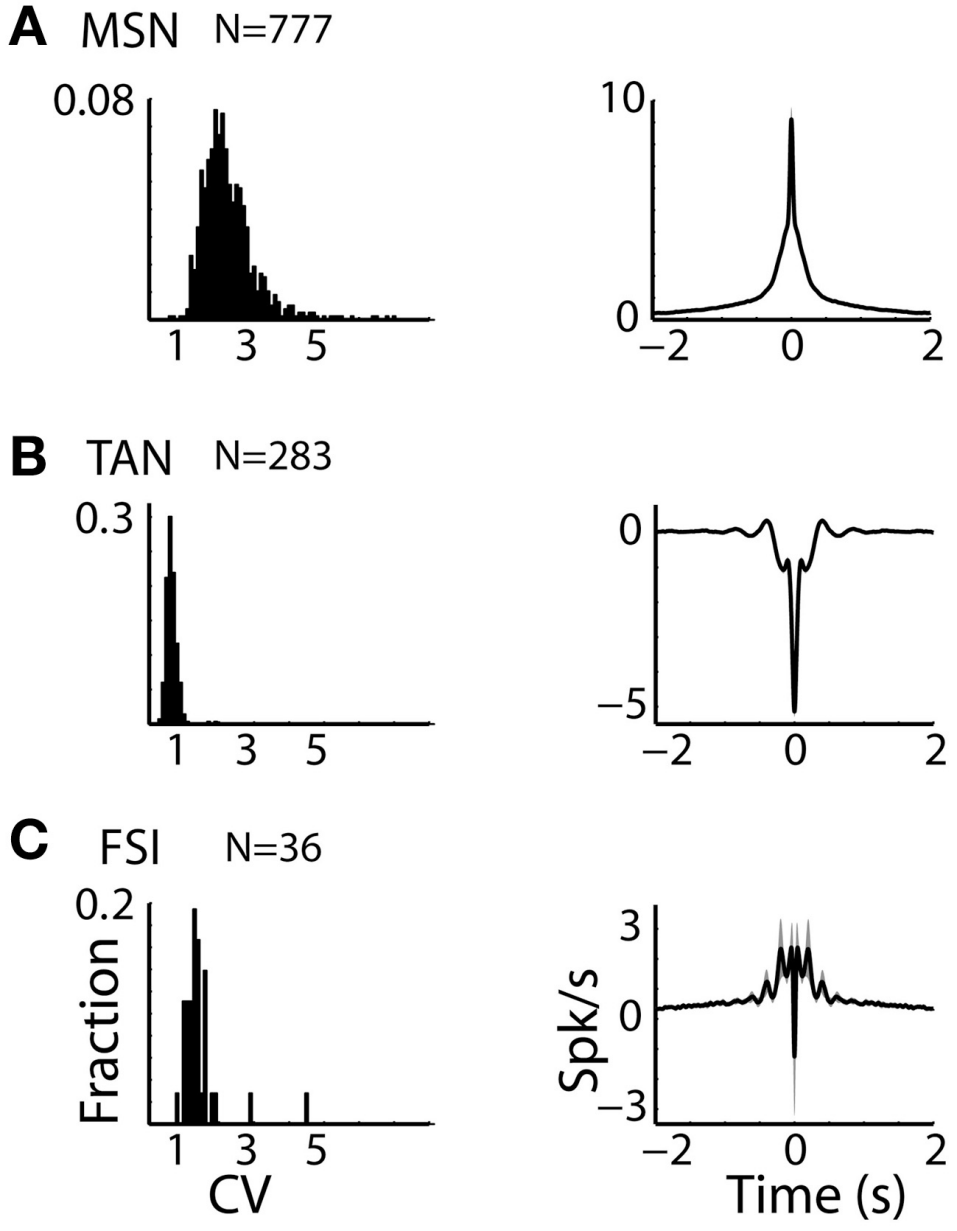
**Striatal MSNs, TANs, and FSIs display different firing patterns. (A)** MSN discharge pattern. Left subplot: Distributions of the CV of the ISIs of striatal MSN neurons. Abscissa: CV. Ordinate: fraction of cells. Right subplot: Average ± SEM (solid line and envelope) auto cross correlation histogram of MSNs normalized by the average discharge rate and averaged over all cells. N is for number of neurons. **(B)** TAN discharge pattern. Same conventions as in **(A)**. **(C)** FSI discharge pattern. Same conventions as in **(A)**.

The discharge patterns of the recorded striatal neurons were characterized by the ISI CV and the auto-correlation histograms (Figure 2). The ISI CV is defined as the SD/mean of the ISIs of each neuron and was calculated on the entire recording epoch (including the ITI). The auto correlation histogram (ACH) was calculated for a delay of 2 s. The ACH of each neuron was calculated for each task event and averaged to provide the raw ACH. The raw ACH was normalized by the average firing rate of the cell. Note that the CV is affected only by the first order ISI, whereas the ACH is affected by all spikes occurring within the 2 s interval.

### Data analysis of single cell responses

**Figure 3:**
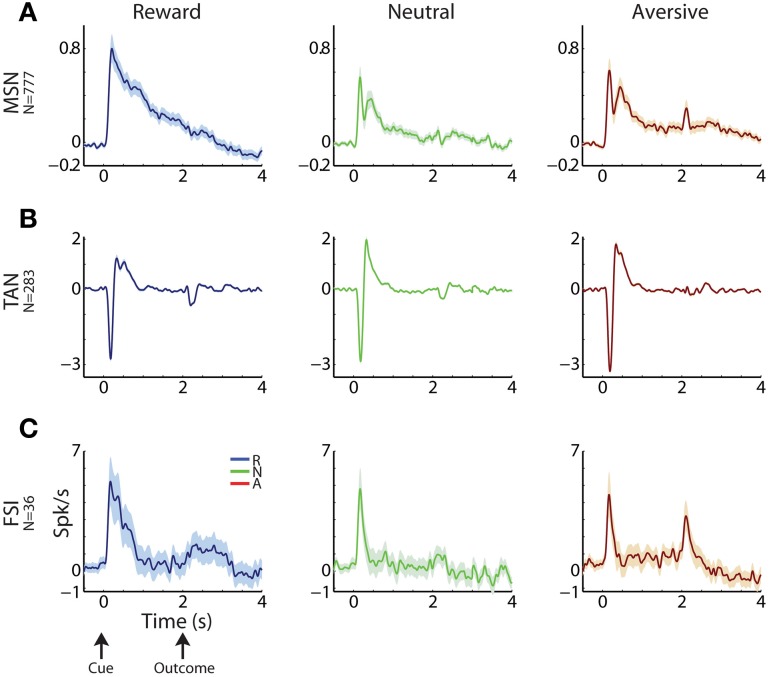
**Striatal MSNs, TANs, and FSIs display different response profiles. (A)** MSN response profile. Average response ± SEM (solid line and envelope) to cue presentation (0 s) and outcome delivery (2 s). Ordinate: firing rate in Hz normalized by the ITI discharge rate. Blue, reward events; red, aversive events; green, neutral events. N is for number of cells. **(B)** TAN response profile. Same conventions as in **(A)**. **(C)** FSI response profile. Same conventions as in **(A)**.

Neural responses to behavioral events were characterized by a post stimulus time histogram (PSTH) starting at cue presentation and ending 2 s after outcome delivery (Figure 3). PSTHs were calculated in 1 ms bins and smoothed with a Gaussian window (*SD* of 20 ms). The baseline firing rate was calculated by averaging the firing rate in the last 3 s of the ITI and was subtracted from the smoothed PSTH. To determine a significant response in a single PSTH, we calculated the SD of the PSTH of the last 3 s of the ITI using the same number of trials as in the studied PSTH and identified time segments in which the deviation from the baseline firing rate exceeded three times the ITI-SD. A response was considered significant only if the duration of the deviant segment was >60 ms (three times the SD of the smoothing filter).

### Data analysis of response similarity of cell pairs

**Figure 4:**
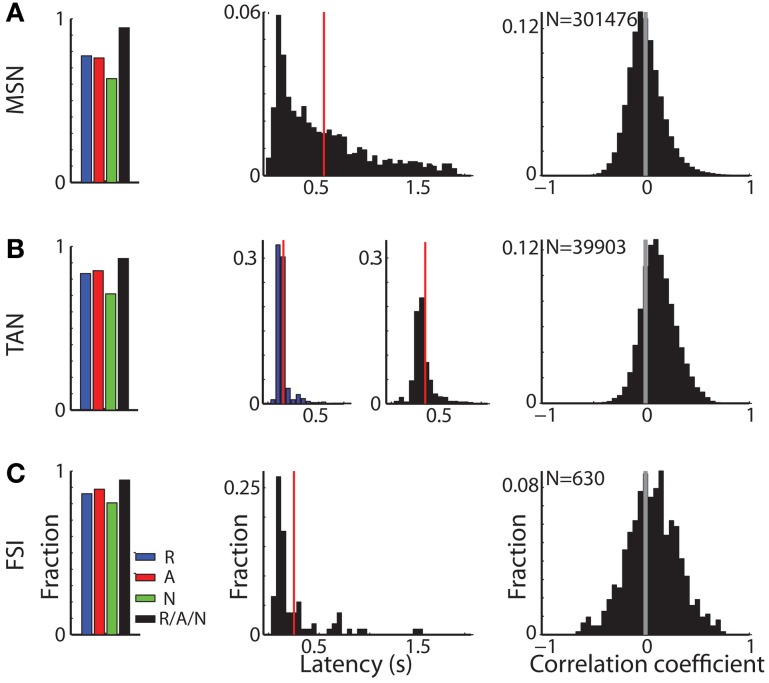
**Different response characteristics of striatal MSNs, TANs, and FSIs. (A)** MSN response profile. Left subplot: distribution of MSNs that had a significant response. Blue, red, and green bars: fraction of cells that had a significant response for reward, aversive, and neutral events, respectively. Black bar: fraction of cells that had a significant response to at least one of the task events. Second subplot: distribution of response onset. Abscissa: time in seconds for significant increase in firing rate. Red line marks the average response onset time. Right subplot: distribution of the signal correlation between all (simultaneously and non-simultaneously recorded) MSN pairs. N is for number of pairs. **(B)** TAN response profile. Same conventions as in **(A)**. In the second subplot: distribution of response onset, left and right columns: latency of significant decrease and increase in firing rate, respectively. **(C)** FSI response profile. Same conventions as in **(A)**.

The signal correlation (Figure 4, right column) was calculated between all cell pairs (simultaneously and non-simultaneously recorded) within each population as described previously (Joshua et al., 2009; Adler et al., 2013). Briefly, a signal correlation measures to what extent a pair of neurons tend to respond similarly to the behavioral events (i.e., similarity of the PSTH vectors). For each neuron we computed the PSTHs in 100 ms bins (without smoothing) for all behavioral events. We combined all PSTHs of a single cell into one matrix with rows for each behavioral event and columns for each 100 ms time bin. For each column, we subtracted that column's mean and then flattened the matrix into a single vector. For each pair of neurons we computed their signal correlation by calculating the correlation coefficient of these two vectors. Signal correlation values range from plus one (for highly correlated response profiles) through zero (non-correlated response profiles) to minus one (anti-correlated response profiles).

### Data analysis of correlative spiking activity of simultaneously recorded cell pairs

**Figure 5:**
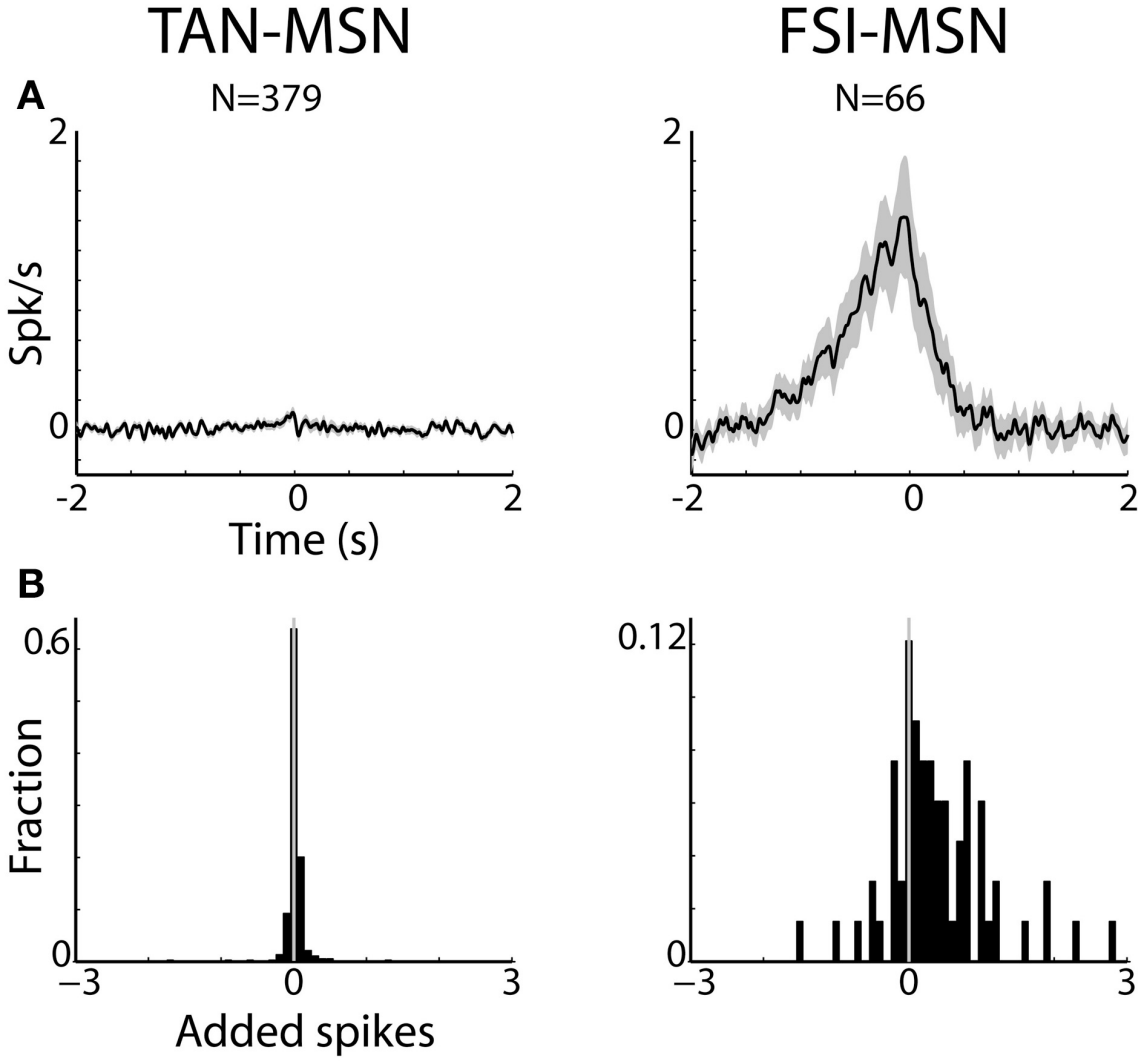
**MSNs are differentially correlated with striatal interneurons. (A)** Raw (with no normalization) cross correlation histograms (CCH) between pairs of striatal interneurons and MSNs averaged over all pairs. Average and SEM; black line and gray shaded envelope, respectively. Abscissa: time in seconds. The MSN (trigger cell) discharge is at time zero. Ordinate: conditional firing rate of the FSI and TAN (reference cell), given a spike of the MSN at time zero. Cross correlation histograms were computed with 1 ms bins for ±2 s around the trigger spike and were smoothed using a Gaussian (*SD* of 10 ms). Left subplot: TAN-MSN. Right subplot: FSI-MSN. N stands for the number of simultaneously recorded pairs. **(B)** Distribution of the average number of added spikes of the reference (MSN) cell in the corrected CCHs around the time window of ±1.5 s. Abscissa: number of added spikes. Ordinate: ratio of pairs (note the different y-scales).

**Figure 6:**
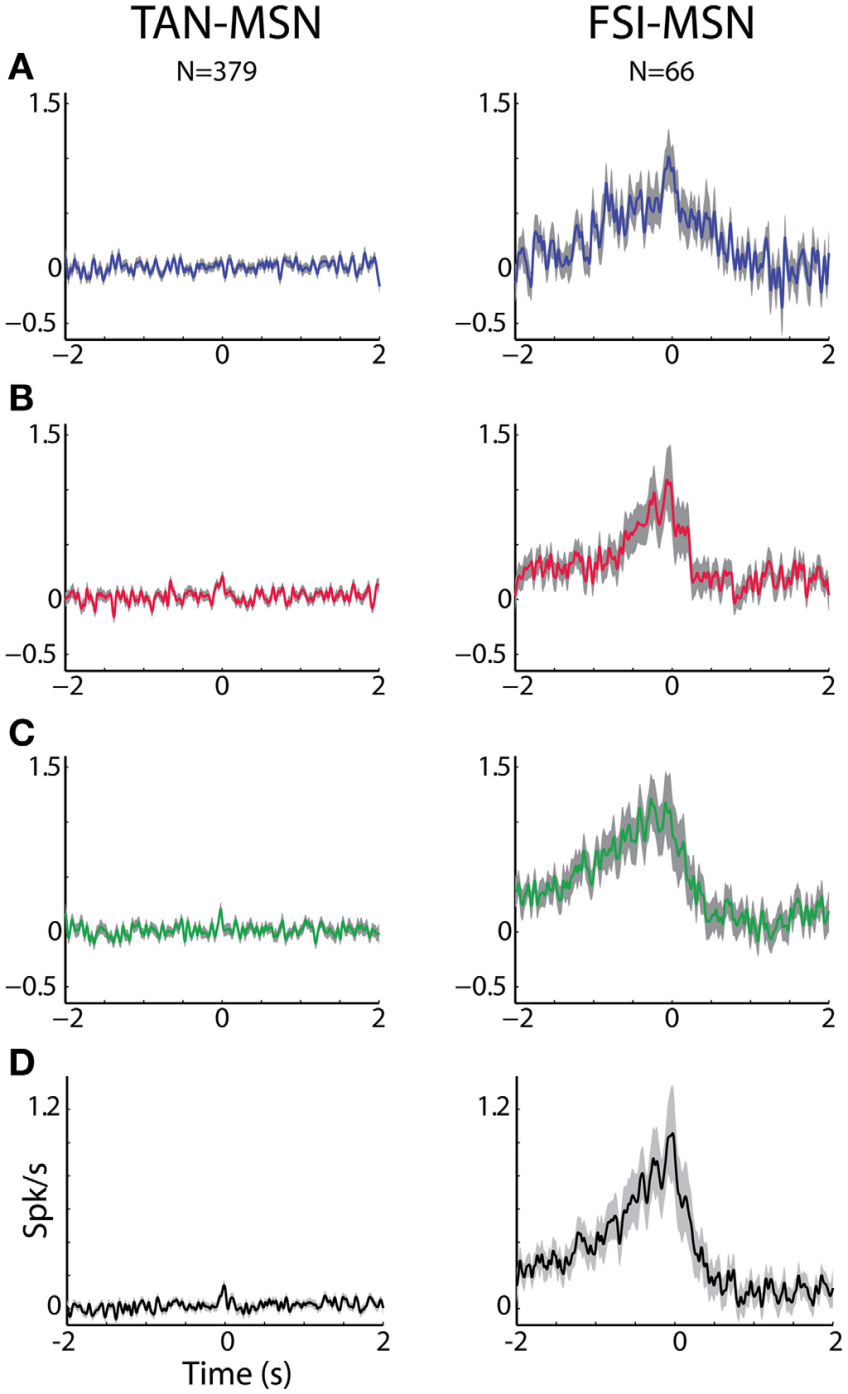
**MSN-FSI and MSN-TAN correlation is not dependent on task event. (A)** Normalized CCH (using the PSTH predictor) averaged over all interneurons to MSN pairs for the reward event. The MSN (trigger cell) discharge is at time zero. Ordinate: conditional firing rate of the TAN or FSI (reference cell), given a spike of the interneuron at time zero. **(B)** Normalized CCH averaged over all interneurons to MSN pairs for the aversive event. Same conventions as in **(A)**. **(C)** Normalized CCH averaged over all interneurons to MSN pairs for the neutral event. Same conventions as in **(A)**. **(D)** Normalized CCH (using the PSTH predictor) averaged over behavioral events for all interneurons to MSN pairs.

**Figure 7:**
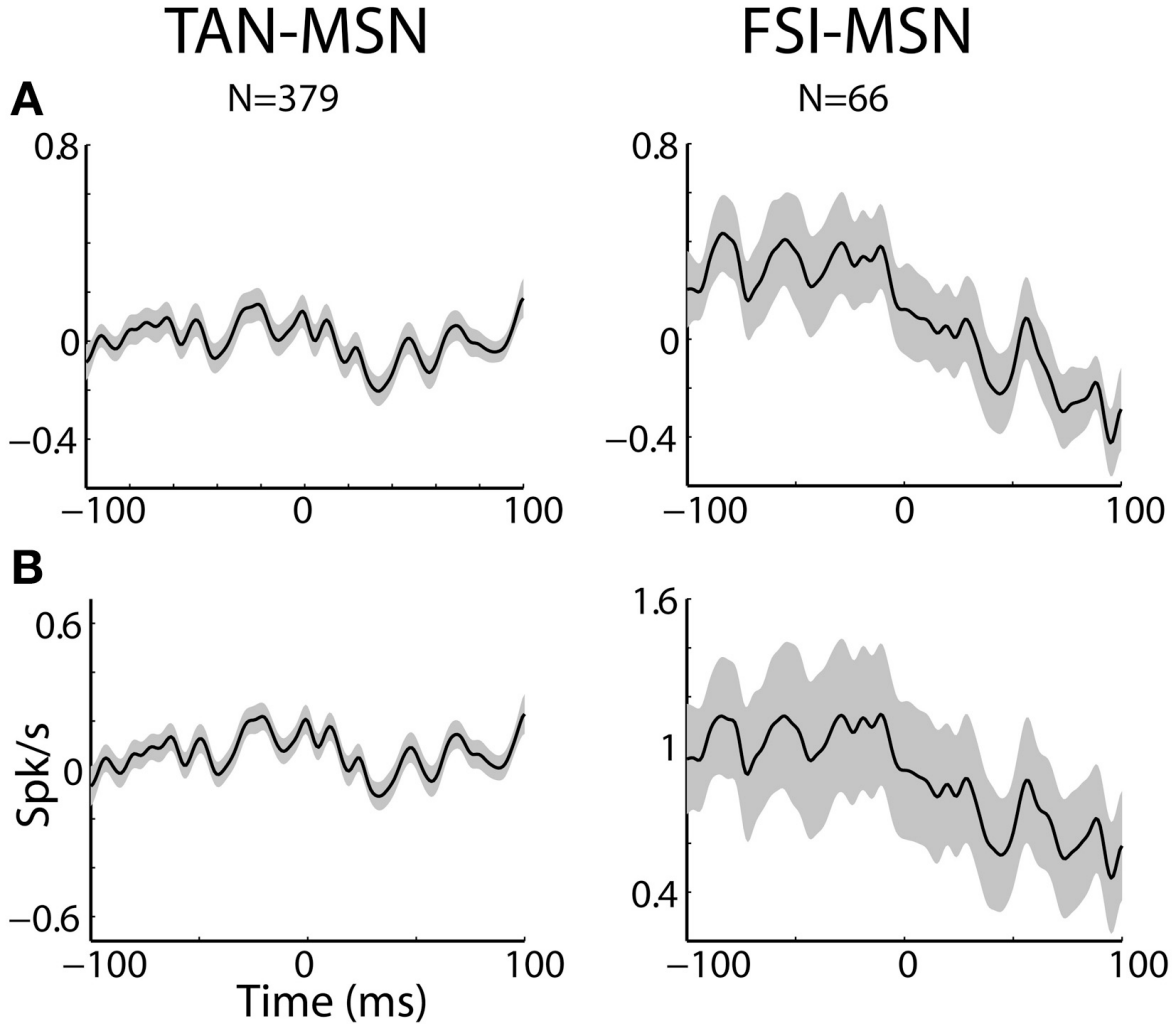
**MSN-interneuron pairs do not display narrow peaks or troughs in their cross correlation histograms. (A)** Raw (with no normalization) cross correlation histograms (CCH) between pairs of striatal interneurons and MSNs averaged over all pairs. Black line and gray shaded envelope display average and SEM values, respectively. Cross correlation histograms were computed with 1ms bins for ±100 ms around the trigger spike and were smoothed using a Gaussian (*SD* of 2 ms). The MSN (trigger cell) discharge is at time zero. Ordinate: conditional firing rate (spikes/s) of the FSI and TAN (reference cell), given a spike of the MSN at time zero. Abscissa: Time lag (in ms) around the discharge of the trigger cell. Axis labels on lower left subplots apply for all subplots. **(B)** Normalized CCH (using the PSTH predictor) averaged over behavioral events for all interneurons to MSN pairs.

**Figure 8:**
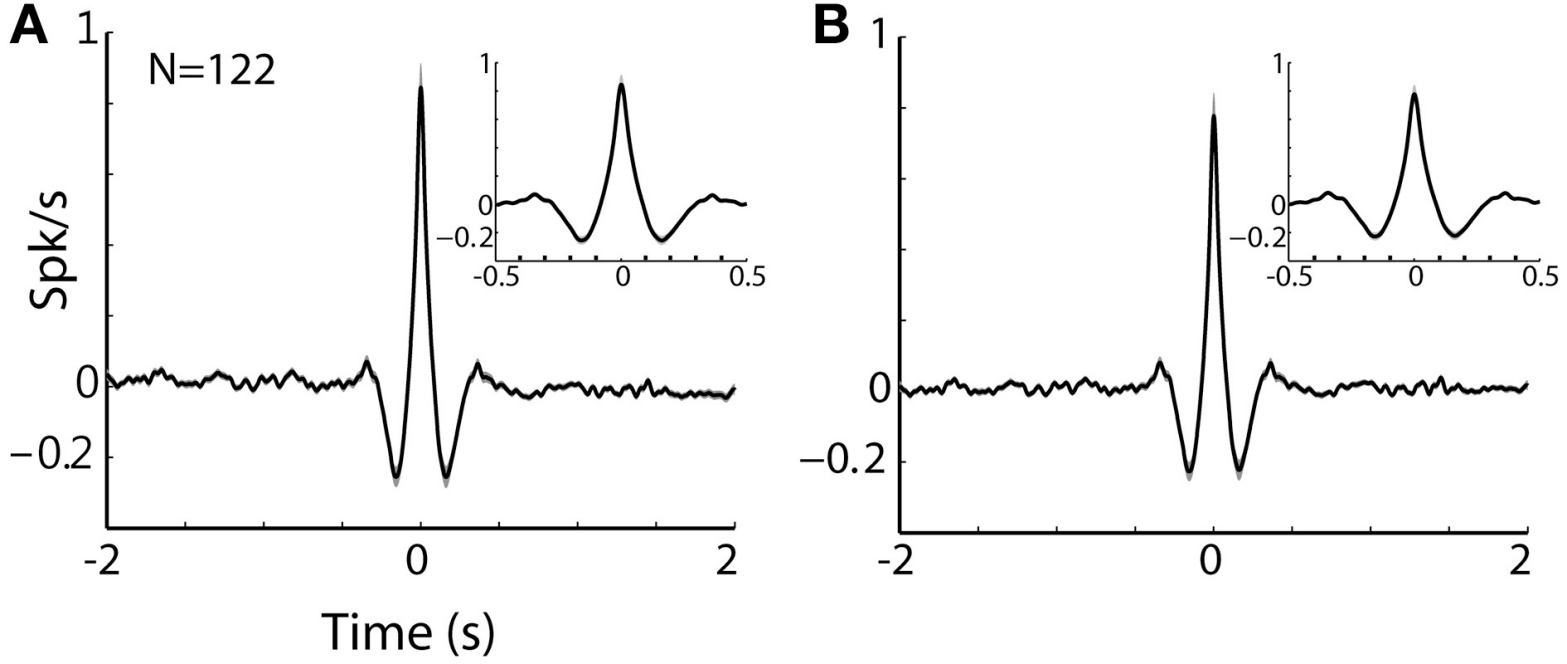
**TAN-TAN pairs display narrow peaks in their CCHs. (A)** Raw (with no normalization) cross correlation histograms (CCH) between pairs of striatal TANs averaged over all simultaneously recorded pairs. Black line and gray shaded envelope display average and SEM values, respectively. Cross correlation histograms were computed with 1 ms bins for ±2000 ms around the trigger spike and were smoothed using a Gaussian (*SD* of 10 ms). Ordinate: conditional firing rate of the reference cell, given a spike of the trigger cell at time zero. Inset: CCH at shorter time scale (±500 ms around the trigger spike). **(B)** Normalized CCH (using the PSTH predictor) averaged over behavioral events for all TAN to TAN pairs.

Spike to spike synchronization (Figures 5–8) between simultaneously recorded MSN-TAN, MSN-FSI, or TAN-TAN pairs was determined using cross correlation histograms (CCHs, Perkel et al., 1967). CCHs were computed with 1 ms bins for ±2 s around the trigger (MSN) spike and the conditional discharge rates of the reference cells (TAN or FSI) were smoothed using a Gaussian (*SD* of 10 ms). For the TAN-TAN pairs the selection of the trigger and the reference cell was done randomly. Only cell pairs with minimal isolation quality (>0.7, Joshua et al., 2007) and rate stability that were recorded simultaneously for more than 21 and 30 min (monkey L and G, respectively) were included in the database. We used different inclusion criteria for the two monkeys in order to have a similar number of trials for each category for the two monkeys (two and three different outcome magnitudes were used in monkey L and G, respectively, see Behavioral task details above).

CCHs were computed separately for each task event and averaged to provide the raw CCH. Raw CCH may reflect the common activation of the recorded neurons either by the intrinsic network connectivity or by the common activation by behavioral events. However, the common activation by the behavioral events could be detected also in trials that have not been simultaneously recorded. Raw CCHs can be therefore normalized (corrected for common modulation of discharge rate) by using PSTH (reflecting the average responses of the recorded cell) and shift predictors (shuffling of the trials). As expected for stationary data, PSTH and shift predictors yielded similar results. Only the PSTH correction method is presented here.

To determine a significant peak/trough in a single CCH we calculated the SD of the last 0.5 s in both negative and positive lags of the CCH, and identified segments in which the CCH (±1.5 s around zero) exceeded three times the SD. A CCH was considered to a have a significant peak/trough only if the duration of the deviant segment was >30 ms (three times the SD of the smoothing filter). We used additional methods (Abeles, 1982a) to determine the significance of the CCH and obtained similar results (data not shown).

To better characterize the CCHs we calculated the area under the curve of the normalized CCH at ±1.5 s time lags. Specifically, we summed the number of spikes in the ±1.5 s CCH time bins and divided this sum by the number of bins to obtain the average number of added spikes of the reference cell (FSI or TAN) around the occurrence of a spike of the trigger (MSN) cell. This parameter ranges from negative values (indicating inhibitory correlation) through zero (indicating no correlation) to positive values (indicating positive correlation).

Finally, to determine the skewness of a significant CCH we calculated a symmetry index. This index is found by subtracting the number of significant bins in the negative lag of the CCH from those in the positive lag divided by their sum.

## Results

### Striatal cell classification and identification

We recorded the activity of striatal neurons from two monkeys engaged in a well-practiced classical conditioning task (Figure 1A). The task involved presentation of visual images (cues) predicting either food outcome in rewarding trials, air puff in aversive trials or neither in neutral trials (Adler et al., 2012, 2013). Recordings were made from two to eight electrodes simultaneously in all striatal domains (anterior caudate, putamen and ventral striatum, Figure 1B). We classified striatal cells into three distinct groups using the waveform profiles (300–6000 Hz band-pass filtered extracellularly recorded activity) and the average firing rates of the recorded neurons (Figures 1C–E). Of the 1287 neurons that passed our inclusion criterion, 777 were classified as striatal phasically active neurons (presumably striatal projection neurons, MSNs), 283 were classified as TANs (presumably striatal cholinergic neurons), and 36 as FSIs (presumably striatal parvalbumin expressing GABAergic neurons). As reported previously in the rodent (Berke et al., 2004; Berke, 2008), the primate FSIs had the narrowest spike waveform lengths and the fastest average firing rates. TANs had the widest spike waveform lengths and intermediate firing rates. Finally, MSNs displayed an intermediate waveform length and the slowest firing rates.

### Distinct discharge patterns of striatal neurons

The three populations of striatal neurons also displayed distinctive firing patterns (Figure 2). TANs had the lowest values of CV of their ISIs with a very narrow distribution, revealing the tendency of these neurons for regular discharge. The CV of the MSNs ISIs was larger and broadly distributed, and the distribution of FSIs CV was intermediate in values and variance (Figure 2, left column).

Similar phenomena were observed in the average auto-correlograms of the three populations (Figure 2, right column). The auto-correlogram reveals the probability of neurons to discharge a spike as a function of time relative to a previous spike (at time = 0) of this neuron. The average auto-correlogram of the TANs (Figure 2B) shows a relative refractory period with a tendency for rebound after discharge. On the other hand, the average auto-correlograms of both the MSN and FSI (Figures 2A,C) revealed their tendency to fire at burst (central peaks in the histograms) that lasted ~0.5 s.

### Distinct population response patterns of striatal cells

Cells in all three sub-populations were highly modulated by the task, particularly to cue presentation (Figure 3). More than 93% of striatal cells (for all three populations) responded to at least one of the task events (Figure 4, left column). However, across striatal sub-populations, the cells displayed distinct response profiles.

MSNs (Figure 3A) typically responded with an average sustained increase in discharge rate to the visual cues, which started on average 547.2 ± 9.8 ms after cue presentation. As reported previously (Adler et al., 2012, 2013), MSNs displayed highly diverse responses. The diversity of the responses of neuronal population can be characterized by the distribution of the signal correlations; i.e., the similarity of the vectors of responses of two neurons of this population (Oram et al., 1998; Averbeck and Lee, 2004; Cohen and Kohn, 2011). The neural activity of MSN-MSN pairs was characterized by a symmetrical signal correlation distribution (average signal correlation ± SEM; 0.004 ± 0.0003, Figure 4A right).

Unlike MSNs, TANs (Figures 3B, 4B) responded with a very stereotyped and synchronized (average TAN-TAN signal correlation ± SEM; 0.12 ± 0.0008, Figure 4B, right) pause and rebound excitation to cue presentation (Figure 3B), which was very sharp and immediate (average ± SEM onset to pause response: 153.2 ± 3.9 ms, to excitation: 334.9 ± 4.9 ms, Figure 4B, 3rd subplot).

FSIs (Figure 3C), like MSNs, responded mostly with an increase in discharge rates to cue and outcome presentation (at time = 0 and 2 s, respectively). However, this response was more immediate than the MSN response (Figure 4C, middle column; average ± SEM FSI onset time: 251.7 ± 27.4 ms, One-Way ANOVA, *p* < 0.05, *f* = 75.13, *df* = 2; MSN response onset time was different from that of TAN and FSI). In terms of similarity of the neural responses of FSI-FSI pairs (Figure 4C right column), FSIs were not as diverse as the MSNs (average signal correlation ± SEM; 0.06 ± 0.009). However, they also did not display the highly synchronized activity pattern of TANs that is characteristic of basal ganglia neuromodulator groups (e.g., dopamine neurons and TANS, Morris et al., 2004; Joshua et al., 2009). A One-Way ANOVA revealed that the distribution of the FSI-FSI signal correlation was different (*p* < 0.05, *f* = 8.78, *df* = 2) from that of MSN-MSN and TAN-TAN pairs.

To sum up, all three populations of striatal projection and interneurons were highly modulated by the task; however, they differed considerably in their response profile and response synchronization levels.

### MSNs are differentially correlated with striatal interneurons

Figures 5A, 6 display the raw and corrected average CCHs between striatal MSN-TAN and MSN-FSI pairs (left and right columns, respectively).

The CCHs between simultaneously recorded pairs of TANs and MSNs were typically flat. In fact, all (*N* = 379 pairs) but three MSN-TAN pairs were not significantly correlated. We further calculated the average number of added spikes of the reference cell (TAN) to the trigger cell (MSN) around the corrected CCH time window of ±1.5 s (see Methods). A negative value would indicate that whenever the trigger cell spiked, the reference cell was more likely to suppress its discharge, a positive value would indicate the opposite, and zero would imply there was no correlation between the two. As predicted by the average flat CCH, we found the distribution of added spikes for the MSN-TAN pairs to be symmetrical around zero and not significantly different from zero (*Z*-test, *p* = 0.7, Figure 5B, left).

To ascertain that the average flat MSN-TAN CCH was not a result of opposing effects canceling each other out, we examined the CCHs separately for each type of task event and normalized them by a PSTH predictor (Figure 6, left). We found the raw (data not shown) and the normalized CCHs were typically flat for all behavioral events. There were no MSN-TAN pairs with significant CCHs for the reward event and only a single pair had a significant CCH for the aversive and neutral events.

Unlike the flat CCHs of MSN-TAN pairs, the MSN-FSI pairs were highly correlated. The average raw CCH of all MSN-FSI pairs (*N* = 66 pairs, Figure 5A, right) displayed a very broad positive and asymmetrical peak. Even after normalizing the raw CCHs by a PSTH predictor (Figure 6, right) to compensate for the effects of similar responses (Figures 3, 4) a broad positive peak remained. Most of the MSN-FSI pairs that displayed a significant CCH (*N* = 29 pairs) had a positive peak (*N* = 24 pairs) and only five had a negative trough. This is evident both in the average CCH (Figures 5A, 6) and in the positively skewed distribution of the CCH number of added spikes (Figure 5B, significantly different from zero, *Z*-test, *p* < 0.05). We found the correlation between MSN and FSI pairs was dependent on the cells' location within the striatum (Figures 1F,G). MSN-FSI pairs with significant correlations were more likely to be located posteriorly (*t*-test, *p*<0.05).

Most MSN-FSI pairs with a significant CCH exhibited an asymmetrical histogram where the peak of the histogram was shifted toward negative values. This implies that the spikes of the trigger cell (MSN) followed those of the reference cell (FSI). We quantified the asymmetry (in the CCHs with significant positive peaks) using a symmetry index (see Methods). Most (20/24) MSN-FSI pairs with a significant CCH had a negative symmetry index with an average of −0.31 ± 0.1 (mean ± SEM, calculated over both positive and negative indices) indicating a CCH peak that was shifted to the left. Like MSN-TAN pairs, the correlation between FSIs and MSNs was not dependent on the task event (Figure 6, right). Furthermore, we examined the MSN-FSI CCHs at shorter time lags of ±100 ms (Figure 7) to search for the expected effects of the mono-synaptic inhibition of MSN discharge by FSI activity. We did not find troughs (or peaks) in the raw (Figure 7A) and PSTH predictor normalized (Figure 7B) CCHs in these time frames (none of the pairs were significant).

## Discussion

We simultaneously recorded the spiking activity of striatal projection neurons (MSNs) and interneurons (TANs or FSIs) from monkeys engaged in a classical conditioning task involving rewarding, aversive, and neutral cues.

All striatal neurons were highly responsive to the behavioral events, but they displayed differential response properties. Striatal MSNs displayed the most sustained (Figure 3) and diverse response pattern (symmetric and broad distribution of the values of MSN-MSN signal correlation; Figure 4, right column). Striatal TANs (presumably cholinergic interneurons) displayed the most transient and synchronized activity pattern (the distribution of TAN-TAN signal correlation was significantly shifted to the right). Finally, striatal FSIs (presumably GABAergic interneurons) displayed intermediate values in both parameters.

The TAN-MSN CCHs were flat, suggesting a modulatory rather than a driving effect of the synchronized TAN activity on MSN neurons. The FSI-MSN pairs displayed a broad and asymmetrical peak in their CCHs. Thus, our correlation analysis of the spiking activity of remote (>0.5 mm) FSI-MSN pairs does not reveal evidence for mono-synaptic inhibition, but it shows that generally FSI discharges precede MSN spikes.

### Striatal MSNs and TANs are not correlated

Striatal TANs constitute a very small percentage of striatal cells (Aosaki et al., 1995). Nonetheless, their widespread axonal field suggests that they should have a significant influence over MSN activity via their muscarinic synapses (Bolam et al., 1984; Bonsi et al., 2011). Anti-cholinergic agents were the first effective pharmacological treatment for Parkinson's disease, and their significant role in the pathophysiology of basal ganglia related disorders is emphasized in the dopamine-acetylcholine balance hypothesis (Calabresi et al., 2006; Aosaki et al., 2010; Sciamanna et al., 2012). Acetylcholine secreted by TANs can affect MSNs directly by changing the cells' excitability (Kreitzer, 2009; Goldberg et al., 2012) or indirectly by altering the dopaminergic input to the striatum (Threlfell et al., 2012). However, striatal TANs (like dopaminergic neurons, Moss and Bolam, 2008; Matsuda et al., 2009; Rice et al., 2011) probably have widespread influences via volume conductance and extra synaptic effects. Thus, they can modulate (in conjunction with the dopaminergic and other modulators of the striatum) the efficacy of the cortico and thalamo-striatal synapses, rather than directly affecting their target neurons' ongoing discharge (Kreitzer, 2009; Higley et al., 2011).

Consistent with this reasoning, we did not find any correlations between the TANs' spiking activity and that of the MSNs (Figures 5–7 left columns), in line with a previous primate study of TAN-MSN correlations (Kimura et al., 2003). In this study, Kimura et al. reported significant (serial) correlation only between 3 out of 16 TAN-PAN pairs (Kimura et al., 2003, last line of their Table 1). Furthermore, we previously reported a lack of TAN—globus pallidus correlations in the normal (before MPTP) primate (Raz et al., 2001). As most of the innervation of pallidal neurons (>90% of their synapses, Percheron et al., 1994) is from striatal MSNs, a lack of TAN-pallidal correlation is congruent with a lack of TAN-MSN correlation.

The finding that MSN and TANs were not synchronized seems to be at odds with a recent optogenetic study (English et al., 2012) revealing strong TAN-MSN poly-synaptic modulation mediated by neuropeptide Y-neurogliaform (NPY-NGF) interneuron. The lack of TAN-MSN correlations is even less-expected given the physiological studies revealing TAN-TAN synchronization (Raz et al., 1996; Kimura et al., 2003; Morris et al., 2004). These correlation studies imply a functional redundancy among TANs (i.e., the ongoing spiking activity of a single TAN is a faithful representation of the entire TAN network). Furthermore, beyond the synchronization of the spontaneous TAN spikes, TANs also show exceptional similarity in their responses to behavioral events. Indeed, many studies of the responses of TANs to behavioral events indicate that the TAN network is globally synchronized (See Graybiel et al., 1994, Figure 4; Adler et al., 2013, Figure 9). Thus, the finding of MSN-TAN flat correlation cannot be neglected on the basis of the spatial distance (>0.5 mm) between the MSN-TAN of this study.

Nonetheless, the practical implication of the physiological TAN-to-TAN synchronization is still quite modest. The typical shape of a TAN-TAN cross-correlogram can be characterized as triangle with a 100 ms base (around time zero, the time of a spike of the trigger TAN) and height of 1 spike/s above the average discharge rate of the reference TAN (See Figures 8A,B for raw and PSTH predicted normalized TAN-TAN correlograms, respectively). Namely, there is increased probability (beyond the baseline discharge rate) for one TAN to emit a spike at 100 ms around the discharge of another spike. This synchronization is thus very different from the optogenetic stimulation which probably induces a considerably stronger and sharper synchronization between TANs. We therefore suggest that the difference in the TAN-MSN connectivity found in our study and the studies that used optogentic tools (English et al., 2012) are due to these different time and intensity scales of TAN synchronization.

### Lack of evidence for mono-synaptic inhibition in the FSI-MSN cross correlation histograms

In this study we used the waveform profiles of the extracellularly recorded spikes and the average discharge rates (Figure 1D) to classify three populations of striatal neurons (MSN, TANs, and FSI). We have found that other parameters (e.g., discharge pattern and responses to behavioral events) also revealed different profiles for the three classes of neurons. Following the rodent literature, we assume that our FSIs are the PV expressing GABAergic interneurons of the striatum. However, TH-expressing neurons can also be fast-spiking and calretinin neurons, which are particularly numerous in the primate striatum, could also make up part of the sample (although we do not yet know their spike shape, they are also GABAergic interneurons). The methodological limits of extra-cellular recordings in behaving animals do not enable us to verify that the FSIs recorded here represent a single type of striatal interneuron, and this should be further clarified by future studies.

*In vitro* studies have demonstrated that single FSI spikes can delay or abolish MSN spikes (Koos and Tepper, 1999; Planert et al., 2010). Furthermore, the FSIs are coupled by gap junctions (Kita et al., 1990; Koos and Tepper, 1999). Together, these properties were interpreted as suggesting that FSIs synchronously inhibit MSNs. However, in line with recent theoretical (Hjorth et al., 2009) and rodent *in vivo* studies (Berke, 2008; Gage et al., 2010; Lansink et al., 2010), we found that in the primate, the FSI population does not show synchronized spiking activity and does not respond similarly to behavioral events (Figure 4C, right subplot). Furthermore, as in these *in vivo* rodent studies (Gage et al., 2010; Lansink et al., 2010) we could not detect narrow troughs in the FSI-MSN CCHs. Finally, our results are in line with an earlier rodent study (Sharott et al., 2009). Although this study was carried out under anesthesia, it demonstrates the lack of negative correlation between MSNs and FSIs and that FSI-MSN correlations are stronger than TAN-MSN correlations.

This discrepancy between *in vitro* vs. the *in vivo* rodent and current primate studies could possibly be rooted in differences between intra- and extra-cellular recording methods. Intracellular studies are biased for adjacent neurons. If the *in vivo* FSI network is not synchronized, the lack of short-latency troughs in the FSI-MSN CCHs may reflect a selection bias for extra-cellular recording of only unconnected MSN-FSI pairs. In fact, the probability of detecting a connected pair with our multiple electrode setup (0.5 mm horizontal distance between electrodes) was small since FSI make strong and dense projections on MSN neurons within a 0.3 mm radius of their soma (Koos et al., 2004; Mallet et al., 2005; Gittis et al., 2010). However, Gage et al. (2010) only examined pairs recorded by the same tetrode, whereas the MSN is most probably within the axonal field of the recorded FSI, and as here, failed to detect evidence for strong functional effects of the mono-synaptic FSI-MSN connection.

FSI-MSN connections show substantial depression during continuous discharge (Klaus et al., 2011). The lack of evidence for monosynaptic inhibition between spikes of FSIs and MSNs could also reflect the high discharge rate of FSIs in behaving animals (unlike slice preparations), thus leading to prolonged synaptic depression of the MSNs (see discussion in Gage et al., 2010). However, see a recent study of local pallidal interactions (Bugaysen et al., 2013) revealing that despite significant short-term synaptic depression and the high frequency discharge of pallidal neurons the local network is modulated by these depressing synapses.

Finally, such discrepancies between *in vitro* experiments (mainly recording intra-cellular sub threshold phenomena) and the spiking activity of pairs of neurons have been reported previously (Abeles, 1982b; Eggermont, 1990; Renart et al., 2010). These may reflect the lower sensitivity of cross-correlation methods for the detection of inhibition (Aertsen and Gerstein, 1985); however, fast inhibition of pyramidal cells by local interneurons has been detected by cross correlations studies in the cortex (Bartho et al., 2004; Gage et al., 2010). In our view, the lack of evidence for short latency depression of MSN discharge by FSI spikes highlights the non-linearity of the input-output relations of the striatal microcircuits (see further Discussion below).

### MSNs and FSIs are activated by a common input

FSIs project heavily onto MSNs. They are highly sensitive to cortical input and display shorter response latencies than MSNs and were therefore suggested to mediate striatal feed-forward inhibition (Tepper et al., 2008). Our response onset measurements (Figure 4, mid column) extend this observation to behaving primates as well.

The MSN-FSI CCHs in this study displayed an asymmetrical broad (~1 s) peak. This very broad peak might explain the discrepancy between our study and a previous rodent study (Gage et al., 2010) which did not find peaks in 100 ms normalized CCHs. The broad CCH peak likely originated from a common input to both cell types. It remains to be determined whether the cortical projection to the FSIs is distinct to a certain extent from other cortico-striatal projections (Berke, 2011). Our data suggest that adjacent MSNs and FSIs receive similar cortical input. This result is congruent with the claim that FSI feed-forward inhibition expands the dynamical range of afferent input to which the MSNs can respond by setting a threshold for MSN activation that is proportional to stimulus strength (Pouille et al., 2009; Gittis et al., 2010). For FSI feed-forward inhibition to regulate the MSN activity dynamic range, FSIs must spike prior to the MSNs in response to afferent stimulation. The asymmetrical FSI-MSN CCHs found in our study (Figures 5, 6 right columns) and the faster onset times of the FSIs (Figure 4, 2nd column) meet this requirement and thus support arguments for faster cortical activation of the FSIs.

The asymmetric MSN-FSI correlograms could also resolve the apparent contradiction between *in vitro* and *in vivo* studies and the lack of evidence for mono synaptic inhibition in the MSN-FSI CCHs in the *in vivo* condition. The latency between FSI firing and MSN inhibitory post synaptic current (IPSC) is very short and the result of the IPSC is often a delaying of MSN spiking. The end result could appear to be MSN firing following FSI firing with a delay that appears to be synchronous (on a large time scale) with the FSI discharge. Our results thus imply that the shared cortical drive to both cell types, the faster responses of FSI and the relative delay of MSN discharge are the main processes in striatal microcircuit physiology. Therefore, consistent with growing evidence (Berke, 2011; Gittis et al., 2011), we suggest that FSIs play a complex and detailed role in modulating MSN activity rather than broad and non-specific inhibition.

Finally, another proposed function of the FSI-MSN synapse is in synchronizing the delayed spikes in MSNs. In future, this could be tested using the Joint-PSTH (JPSTH) method (Aertsen et al., 1989) between two MSN and a FSI recorded simultaneously, and by using the spike of the FSI as the JPSTH trigger. The scarcity of FSI recordings and the low discharge rate of striatal MSN does not enable us (with our current methodological limits) to reliably perform this analysis on our data.

## Concluding remarks

We presented a differential functional relationship between MSNs and two types of striatal interneurons: TANs, the presumably cholinergic interneurons, and FSIs, the presumably PV expressing GABAergic interneurons. We did not find evidence for direct monosynaptic interactions between the MSNs and either striatal interneuron at the level of cross correlation of their spiking activity. However, the flat CCHs of MSN-TAN pairs contrasted with the asymmetric broadly peaked CCHs of MSN-FSI pairs. This suggests that the two interneuron populations play a different role in modulating MSN activity and striatal information processing (Szydlowski et al., 2013). In this report we do not present any data regarding direct cross-correlations between FSI and TANs. This is due to the scarcity of such simultaneously recorded pairs in the striatum of behaving primates. Nevertheless, the robust differences between the correlation patterns of MSNs with TANs and FSIs suggest that these striatal interneurons have independent and different functions. Whereas the highly synchronized TANs are likely to have a widespread influence via volume conductance, the less-synchronized FSIs appear to be more involved in spatially constrained feed-forward information processing in the striatal network.

### Conflict of interest statement

The authors declare that the research was conducted in the absence of any commercial or financial relationships that could be construed as a potential conflict of interest.
